# A New Era in Mental Health Care in Vanuatu

**DOI:** 10.1155/2011/590492

**Published:** 2011-04-05

**Authors:** Jill Benson, Dimity Pond, Michelle Funk, Frances Hughes, Xiangdong Wang, Len Tarivonda

**Affiliations:** ^1^Health in Human Diversity Unit, Discipline of General Practice, University of Adelaide, South Australia, Australia; ^2^Pacific Islands Mental Health Network, Port Vila, Vanuatu; ^3^Discipline of General Practice, University of Newcastle, Newcastle, NSW, Australia; ^4^Mental Health Policy and Service Development (MHP), Department of Mental Health and Substance Abuse, World Health Organization, Geneva, Switzerland; ^5^AUT Auckland & UTS Sydney, WHO PIMHnet Facilitator, Wellington, New Zealand; ^6^Regional Adviser in Mental Health, WHO Regional Office for Western Pacific Region, Manila, Philippines; ^7^Department of Public Health, Ministry of Health, Port Vila, Vanuatu

## Abstract

Inequity in health-care delivery for those with mental illness is widespread throughout low- and middle-income countries. In the Pacific Island countries there are many barriers to addressing the growing mental health burden. In an effort to address this problem, the WHO is coordinating the Pacific Islands Mental Health Network involving 18 countries in the Pacific region with the financial support of New Zealand Aid (NZAid). JB and DP have developed and presented mental health training to health professionals, community leaders, and social service personnel in an environment in Vanuatu that is very different from that of their usual Australian-based general practices. They discuss evidence for their work, an outline of the programme, some difficulties working across different cultures, and the enthusiasm with which the training has been greeted. Vanuatu is now well on its way to addressing the inequity of access to mental health care with a culturally appropriate and self-sustaining mental health workforce.

## 1. Introduction

In most high-income countries access to good quality, evidence-based health-care continues to improve. Because of the overwhelming burden of disability caused by mental health problems throughout the world, many high-income countries have begun to address the inequity of access to mental health care with improvements in policies; training; work-force; primary, secondary, and tertiary services; programmes; and public education. As a recent WHO report concludes, people with mental health conditions are among the most marginalized and vulnerable groups. It is important that they be empowered to reach their goals and participate fully in society [1]. 

Governments in many developed countries are recognizing the importance of integrating mental health into primary care services and general hospitals, as well as providing public education, and protecting and promoting the human rights of people with mental disabilities. 

However for those in many low- and middle-income countries (LAMICs), globalization, urbanization, and rapid social change are part of a slowly worsening profile for mental health and the resources for the management of these problems do not match the increased need [2]. Experience in China, India, and Eastern Europe has alerted health professionals to the importance of preparing the health workforce in countries where the traditional culture is being replaced by a more “modern” Westernised culture. For many LAMICs there are many barriers to making good quality mental health care available and until this is addressed the disability and suicide rates due to mental health problems are likely to increase. In some LAMICs there has been an increase in tertiary-level mental health facilities which will only be available to very few people because of access and affordability. Making mental health care available to all should also involve primary health care practitioners, traditional healers, teachers, police, corrections, and community leaders—a whole-of-community approach that reflects the complex nature of mental health aetiology and management. 

Vanuatu, with a population of about 220,000 people, has 22 doctors and 450 nurses spread over 83 islands. This very small health workforce mostly operates at a primary care level; there are no full-time publicly available mental health professionals and only a few people with any mental health training at all. Building up, supporting and mentoring a mental health workforce, reducing stigma in the community, training the informal mental health workforce, and advocating with Government and NGOs are all important aims of the Pacific Islands Mental Health Network (PIMHnet), a WHO initiative funded by NZAid.

JB and DP, both General Practitioners (Family Physicians) in Australia, have developed and implemented intensive 3-week mental health training programmes to doctors, nurse practitioners, nurses, NGO workers, teachers, police, corrections, public health officers, and nursing lecturers over 2009 and 2010. The training has included case studies drawn from the experience of the group, an emphasis on primary health care skills, supervised visits to local villages, and the hospital and simple research techniques. In 2010 JB visited many of these professionals in their place of work, often on remote islands, to supervise, mentor, and further train them. Further visits in 2011 will aim to consolidate skills so that more health professionals can be trained and there is a strong network in the country of support for each other.

## 2. Global Mental Health

Poverty, lack of education, unemployment, conflict, disasters, and gender imbalance are well known as the social determinants of mental health [1, 3]. Mental disorders account for 8.8% of the total burden of disease in LAMICs [4] and the additional risks of urbanization, loss of tradition, loss of subsistence farming activity, and loss of extended family mean that those who live in countries in economic transition such as the Pacific Islands are likely to have a worsening mental health profile [2, 3]. Any figures on mental health do not take into account the disability experienced within the family unit, their community, or their country because of the mental illness [3, 5]. The treatment gap for serious mental health disorders in LAMIC is 76–85%, and even for those who are treated, it is often sporadic or with inadequate doses of outdated medication [1].

As deaths decrease from infectious diseases such as measles, pneumonia, gastroenteritis, tuberculosis and malaria, there has been an increase in suicides and alcohol-related deaths in countries such as Eastern Europe, India, South America, and China [2, 3, 6]. Yet many governments of LAMICs have been slow to prioritise mental health. The few resources that are available are often spent on tertiary institutions that are inaccessible for the majority of the population [7]. NGOs and governments need to work together to ensure that mental health is given priority and that this is embedded in the primary care workforce [7]. One-off Western-style training has not been found to be successful, whereas it is both efficient and cost-effective to train primary care workers in a culturally appropriate manner in psychological and pharmacological interventions and then to ensure that they are supervised and supported [7, 8]. Such a strategy will not only decrease morbidity, comorbidity, and mortality but can also encourage resilience, problem-solving, and social support and hence improve the prevention of mental health disorders and decrease referral to high-cost tertiary institutions [6]. Including NGOs, community organizations and traditional resources such as chiefs, traditional healers, and religious pastors as part of mental health training will ensure a more holistic and sustainable approach for addressing mental health problems [1, 5].

The World Health Organization (WHO) and World Organization of Family Doctors (WONCA) recently collaborated to write a report entitled “Integrating mental health into primary care. A global perspective” that sets out “Seven good reasons for integrating mental health into primary care” and “10 Principles for integrating mental health into primary care” (see boxes herein after) [8].

**Box 1**

Seven good reasons for integrating mental health into primary care are as follows.

1. The burden of mental disorders is great.
2. Mental and physical health problems are interwoven.
3. The treatment gap for mental disorders is enormous.
4. Primary care for mental health enhances access.
5. Primary care for mental health promotes respect of human rights.
6. Primary care for mental health is affordable and cost effective.
7. Primary care for mental health generates good health outcomes.

**Box 2**

 10 Principles for integrating mental health into primary care are as follows.

1. Policy and plans need to incorporate primary care for mental health.
2. Advocacy is required to shift attitudes and behaviour.
3. Adequate training of primary care workers is required.
4. Primary care tasks must be limited and doable.
5. Specialist mental health professionals and facilities must be available to support primary care.
6. Patients must have access to essential psychotropic medications in primary care.
7. Integration is a process, not an event.
8. A mental health service coordinator is crucial.
9. Collaboration with other government nonhealth sectors, nongovernmental organizations, village and community health workers, and volunteers is required.
10. Financial and human resources are needed.

## 3. The Pacific Islands Mental Health Network (PIMHnet)

The need for more mental health services in the Pacific region grew out of a situational analysis conducted by WHO which showed that most Pacific nations had growing rates of mental health problems (including suicide) but had few resources to tackle the burden [9]. PIMHnet was formally launched in March 2007 in response to requests by Ministers of Health of Pacific Island countries (Meeting of Pacific Islands Ministers of Health March 2005 Samoa). Its vision is that the people of Pacific island countries will enjoy the “highest standards of mental health and well-being through access to effective, appropriate, and quality mental health services and care”. Its mission is to “facilitate and support cooperative and coordinated activities within and among member countries that contribute to sustainable national and subregional capacity in relation to mental health” [10].

In consultation with its 18 Pacific Island members, several priority areas were identified which needed to be addressed:

- human resource development and training,
- service development focused on primary care,
- policy and planning,
- law reform,
- advocacy.

Since its inception three countries have mental health policies in place with another nine having draft policies. Two countries have new mental health laws and another five have begun planning. Thirteen countries have developed plans for human resource development and training and seven of these are already implementing them. These processes are not sequential and so, for instance, in Vanuatu, the implementation of the human resource development and training plan proceeded prior to the formal adoption of the mental health policy and strategic plan. More information on the activities of PIMHnet can be found at http://www.who.int/mental_health/policy/pimhnet/en/index.html.

## 4. Situation in Vanuatu

Vanuatu is defined by the UN as a “Least Developed Country” with approximately 75% of the population living in rural areas [11]. Accessing health services is very difficult for most people because most of the health services are in cities and large villages and there is a wide distribution of the population and often difficult terrain [12]. As with many LAMICs there is a migration of healthcare workers out of the country, primarily to Australia and New Zealand, and training in any specialty area including mental health is generally conducted overseas with the assistance of fellowships.

The health services in Vanuatu are divided between the Northern Health Group which has a main referral hospital at Luganville on Espiritu Santo and the Southern Healthcare Group with Vila Central Hospital on Efate Island. Each hospital has two mental health beds and a secure “safe room”. There are 5 smaller provincial hospitals; 29 health centres, each with a nurse or nurse practitioner; 74 dispensaries, each with a nurse; and 123 health aid posts and 6 mobile clinics, each with a community worker who does health promotion [12].

Vanuatu has been a subsistence society with a culture of traditional problem-solving through chiefs in each village. There are over 110 different “mother-tongues” spoken in the country and these are the languages where the words for mood, philosophical concepts, and deeper traditional ideas are found. The common language of Bislama is a trade language with no direct translations of any of the Western words for mental health such as depression or anxiety and only a limited mood vocabulary. Social problems have always been taken to the chief to resolve, mostly in a fairly autocratic manner but each chief has had a different style and collaborative approach. Human rights issues for people with severe psychosis depended on the support of the community, the family, how dangerous they were, and what contact they had had with the criminal justice system. People with severe untreated schizophrenia, bipolar disorder and brief psychotic disorder have been given traditional medication and religious exorcism and cared for by their family and community. Women tended to move to their husband's village and look after the extended family, usually sharing gardening tasks with their husband. Life has revolved around traditional ceremonies, tending the garden, caring for the houses and village and family issues. 

As subsistence farming has decreased, young men have moved to the cities to drive taxis and try to get jobs. This urbanization and selling of land has broken many of the family traditions, increased poverty, increased unemployment, decreased social support, and so forth. Young people in the cities are bored and have increased substance misuse and interactions with police. Women are now more educated and want to choose between education, career, marriage, and children. Many people do not want the chiefs to make decisions on their behalf. The chiefs themselves are often feeling overwhelmed with the stress of their positions and the change in their status and are at risk of depression. Vanuatu has a strong religious background with 92% of them Christians. The church and pastors have always had a very important role in counseling and problem-solving for those in their congregation and have mostly been the only source of mental health care available for many people. However a recent survey by a psychiatrist from Australia found that there was a great deal of misunderstanding about mental illness both in the church leaders and in the congregations. 45% of those surveyed thought that mental illness was a sin, 34% due to demon possession, and 38% due to a curse [13].

As the country changes, the health system must change with it to take into account the new illnesses that are likely to emerge. Based on prevalence rates from the World Mental Health Survey in 2004, it is estimated that 13% of the adult population are likely to have a mental health disorder, 3% of these severe [12]. However in 2007 only 48 people in Vanuatu were recorded to have received treatment for a mental disorder [12] indicating a treatment gap of over 99%.

## 5. Vanuatu Training Programme

JB and DP, two general practitioners from Australia who have an interest in mental health, were asked by the WHO to develop and present a programme for PIMHNet over 3 years. This involved developing a culturally appropriate mental health training programme, training key health, and social service personnel; supervising those who have been trained in their places of work; mentoring and setting up support networks for them; presenting more advanced training; and training local doctors and nurses to be mental health trainers and mentors themselves. JB and DP also spent time networking with the formal and informal mental health stakeholders in Vanuatu, including hospital administration, government, church organization, and nongovernment organizations.

**Box 3**

Training visits for health workers and the community are as follows:

- *first visit*: nurses from Vila Central Hospital (4), NGO workers (3), nursing lecturers from Vila (2), military medic (1), government health administrators (2), and doctors from Vila Central Hospital (2);
- *second visit*: 8 nurses, nurse practitioners and nurse aides from outlying islands (three nurses from Santo, one nurse from Pentecost, one nurse practitioner from Tanna, one nurse practitioner from Malekula, one nurse from Torba, one nurse's aid from the local village of Erakor), and 5 social services personnel (one nurse who works in administration in the Department of Health, a chief from an NGO on Santo, a senior Education department administrator, a Correctional Services officer, and the Senior Sergeant of Police); in every session there was someone trained during the first visit to assist with the training; they had done a two-day “Train the trainer” course before the start of the training;
- *third visit*: visiting all the doctors, nurses, and social services personnel in their workplaces; discussing cases, giving additional training, mentoring, seeing difficult patients, and training those people the nurses had trained.

**Box 4**

Programme of presentations for PIMHnet in Vanuatuare as follows:

- what is mental illness?
- depression,
- anxiety,
- communication skills,
- structured problem-solving,
- cognitive behavioural therapy,
- narrative and family therapy,
- alcohol and substance abuse,
- risk management and crisis intervention,
- psychosis and bipolar disorder,
- personality disorders,
- child mental health,
- adolescent mental health,
- mental health and the elderly,
- research skills.

During the first visit to Vanuatu for training, a combination of teaching methods was used. The mornings were taught using an interactive didactic format and the afternoons in workshop style. In the afternoons participants brainstormed topics in groups, role-played what they had learnt in the morning, and refined skills in small group discussions. The content of the case studies and role-plays were built up over the course of the training as JB and DP developed an understanding of the problems that caused mental health issues and how they might present in Vanuatu. Many of these were very different to those in Australia—for instance, the use of kava causing social disruptions in families, the strong religious culture in the community, the role of chiefs in resolving family and community issues, arranged marriages, the importance of making “lap-lap” (traditional dish made with ground taro or yam roots), young men coming to Port Vila to find work, the controversy about the use of marijuana, and so forth. 

Because of the importance of keeping the presentations relevant, culturally appropriate and in-tune with the needs of the participants, most of the sessions were prepared during the first visit to Vanuatu. Both JB and DP have had about 30 years of experience as General Practitioners (Family Physicians) in Australia.

Many resources were used from the WHO but other important ones included JB's book on “Mental Health Across Cultures” [14], “Where there is no Psychiatrist” by Patel [15], and “Essential Skills for Mental Health Care”—a handbook from Ghana [16]. 

The training sessions during the second visit were more difficult as the group that had been identified for training comprised some of the most highly trained nurse practitioners in Vanuatu and others who had no health background or experience at all. The first day was spent discussing the challenge of how to prevent the disability associated with mental health problems becoming so severe that the only avenues available to manage the situation were hospitals, police, corrections, church elders, and so forth. This was a very useful discussion because it enabled this very diverse group to focus on a common goal. Even though they came from such different backgrounds, after about the third day, the differences had almost disappeared and everyone seemed to be working at a similar level. One of the differences between the participants was the issue of clinical care, something the nurses were very familiar and comfortable with. The social services personnel brought a different perspective to many of the sessions because they insisted on discussing the philosophy behind strategies such as confidentiality, parenting, the role of spirituality in mental health, and human rights. 

As with the first training group, the postworkshop questionnaire showed that everyone had enjoyed the course very much, that they thought it was relevant to their current situations, that it had changed the way they related to patients with mental health problems, that their confidence in diagnosing and treating mental health problems had increased because of the course, and that they could help people with mental health problems have better lives. Most thought they could make a management plan for people with depression, anxiety, suicidal risk, crisis, or substance abuse but still felt tentative about being able to develop management plans for those with schizophrenia, bipolar disorder, or somatisation. 

The practical nature of this training was very different to the mental health training that some of the participants had experienced in the past. Previous training was theoretical, with an emphasis on written material and DSMIV and ICD10 diagnoses, and as a consequence health workers were not able to apply any new knowledge or skills in the clinical setting. As General Practitioners, JB and DP were able from the outset to emphasise the acquisition of skills that were practical and relevant. For instance, participants were encouraged to grade patient's illness on a Likert scale from 1 to 10 but also to scale the disability on a scale from 1 to 10. They were then able to ask what would make the patient better or worse, prepare an appropriate risk management plan, and mobilize the patient's resources. Drawings such as the “social atom” to discuss social structure and family trees for children and the “hand exercise” that helps access social and personal assets were very popular. The use of stories and humour and discovery of resilience in Narrative Therapy and the “Cultural Awareness Tool” which helps a health professional find the meaning of a situation for the patient were thought to be two particularly useful tools [14]. These are tools that help explore the patient's understanding of their beliefs and access their own resilience. The Cultural Awareness tool is a series of patient-centred questions, and in Narrative therapy the problem is externalized and the patient is more able to access their own resources for dealing with their problems. They are especially useful when patient and health professionals come from different cultural backgrounds. Respect for the diverse traditional, cultural, and religious beliefs in the group was also extremely important but challenging and using differences in beliefs as problem-solving exercises proved the immediate relevance of the use of this tool. Slow breathing and relaxation techniques were practiced every day until everyone in the group felt confident that they could teach these techniques to patients with panic and anxiety disorder. 

As the skills of the groups improved, the cases developed for the training became more realistic and difficult and they were able to revise and consolidate their knowledge and develop and communicate management plans for the role-playing cases. DP spent time with the first group on her second visit, consolidating their skills and exploring new concepts and strategies with them. This deepened their understanding and improved their confidence in using techniques such as CBT. Only the nurse practitioners and doctors in the group (4 people) were able to prescribe medication; so the emphasis in the course was on diagnosis, assessment, and psychotherapy.

## 6. Supervision and Mentoring

The third trip involved JB and the main mental health nurse from Vila Central Hospital travelling to five different islands, some of them very remote with limited electricity, accessibility, or support for the few health professionals there. Seeing people in their own communities was found to be of the utmost importance. It helped with skill development; it raised the profile of mental health and encouraged local people and other health professionals to trust the nurses who had been trained; it gave the nurses confidence that they were on the right track; it highlighted the importance of mental health being embedded in the primary health care model. 

The list of mental disorders was almost identical to that which a GP in Australia would see, except that most of them had no previous treatment. Because there are no words for mental health in Bislama, clinics were advertised as “Sik long bran” (Brain clinics) and so physical illnesses which can affect mental health were seen such as grand mal seizures, petit mal seizures, temporal lobe epilepsy, parkinsons disease, anaemia, vitamin B12 deficiency, hyponatraemia, liver disease, sequelae of encephalitis, sequelae of cerebral malaria, hypertension, and migraine. All of these except hypertension were clinical diagnoses as there were absolutely no facilities for testing or referral. The rest of the patients had depression, anxiety disorders, bipolar disorder, schizophrenia, substance abuse, personality disorders, and so forth. The only other difference was that of “brief psychotic disorder” which is rarely seen in developed countries [17]. This is often associated with religious experiences as JB found in Vanuatu and lasts less than a month with a return to premorbid functioning. 

Western-based ideas of confidentiality, screening tools, pattern recognition, appropriate places for consultations, management plans, and follow-up all needed to be modified to suit the environment. Consultations were held in many diverse places such as in the grass huts that served as remote nurse aid posts, in patient's houses, in the centre of the village, and in more formal settings in the hospital. Almost all patients came with family or community members, with up to ten people accompanying very sick patients with schizophrenia. Sometimes two local interpreters were needed as patients did not speak Bislama but only the local mother tongue. 

In every area the focal nurses, and nurse practitioners had trained other nurse practitioners, nurses and nurse aids in basic mental health awareness, diagnostic techniques, and treatment skills. They had also been considerably active in raising awareness about mental health problems in their communities. Everywhere the team went there were more people than could possibly be seen during a short visit. Many of the patients had been identified by the nurse aid who had been trained by the nurses that JB and DP had trained. In some places the local chief accompanied the patient and identified other patients who needed to be seen, and others responded to signs or announcements in church. 

All of the “untreated” patients with very disruptive psychosis had been treated with custom medication and had a traditional understanding of what was causing the problem. One of the most powerful health promotion tools was to say that people all over the world have this same disease with very similar symptoms and that this is an illness and not black magic. Untreated psychosis is an enormous burden and worry for families and there is a great deal of stigma associated with the community thinking there is a curse on the person. 

Many of the nurses in remote areas were feeling quite lonely in their quest to diagnose and treat mental health problems and were very pleased to have someone to listen to their successes and problems. Working through case studies and actually seeing patients together was a fertile ground for further training and such supervision is invaluable.

Because the participants of the training on mental health worked in very different workplaces, they found that different skills were useful for them. So, for instance, the nurse in Emergency at Vila Central Hospital had enormous success with using the slow breathing techniques for people who were coming in regularly with “asthma attacks” and had decreased the number of presentations dramatically. The chief had used structured problem-solving to change the way he dealt with the people who came to see him. The nurse in the paediatric ward had used family therapy a great deal and wanted to expand her understanding of this skill. One of the doctors had found it very useful to be able to properly assess the risk of suicide and use some CBT techniques. She had also started to use medication for more people, as she felt more confident about it. One of the remote nurses had found simple narrative therapy very useful and had taught the nurses aids in the area basic diagnostic skills, the hand exercise, slow breathing, and narrative therapy skills. Another remote nurse had started a “mental health clinic” one afternoon a week and had the Likert scales for disease and disability and the “hand exercise” in every patient's notes. He had taught the two other nurses who worked with him so that they were also now doing this. The nurse in Vila Central hospital who had done further training in Australia was seeing a lot of people with schizophrenia and was doing a full mental state examination on all these patients which gave him a clearer picture of the their mental state. The police and corrections people saw a lot of people with substance abuse problems and found the assessment tools and motivational interviewing very useful. 

All of these practitioners were very enthusiastic about having more training as they were often seeing rapid changes in the attitudes, functionality, health, and social circumstances of patients who had previously been struggling with mental health problems but had not been able to alter the situations they were in. All the participants were eager to teach their colleagues what they had found useful and some of those who had been taught in this way had begun to teach others. 

As well as the training, JB and DP spent time networking with mental health stakeholders in Vanuatu, advocating with the government for more resources in mental health, mental health promotion, liaison with churches, development of templates and questionnaires for use in the communities and in hospital, and so forth. By the second visit in 2009, the Vanuatu Mental Health Policy and Strategic Plan (2009–2014) had been approved and many of the students were actively using the techniques and instruments to assist people with mental health problems in the community and the hospital. An Australian volunteer psychologist had been recruited and based in the community and support had been gained from chiefs, the church, and other NGOs to assist with mental health problems in several villages. The hospital was proceeding with delivery of mental health care and is working on a process to facilitate communication between the hospital and the community. Education in mental health was proceeding in other areas such as the military, police, and education department. 

Teaching evaluation and basic research skills was seen to be an integral part of the training as well as the importance of evaluating any programme so that its usefulness could be assessed and lessons learned. The groups discussed the importance of ethics and confidentiality and validated the translations of research tools. 

The Kessler 10 (K10) has been translated into many languages but not into Bislama [18]. After much debate in the group, three different translations were made. The debate highlighted the value of a formal validation process if the K10 was going to be used as a research tool, the caution needed when using such a tool to screen people for mental health problems, and the importance of using clinical judgement rather than a tool to make a diagnosis of mental illness. Patients in the hospital and people in several of the surrounding villages were assessed using these versions of the K10. None had been given a medical diagnosis of mental illness and none were currently being treated for psychological issues, stress, or mental health problems. The collation and analysis of the results was a way of helping health workers understand simple mental health assessment procedures, statistical, and research techniques but lacked the thorough methods required for rigorous research. Of the 80 people (40% male, 60% female) interviewed at Vila Central Hospital, 25% had “severe mental health distress” (defined as a K10 score of over 30). Particularly vulnerable groups were women in the antenatal clinic (30% scored over 30) and men in the 40–49 year age group (75% in the severe range). Comments from learners included a remark that most people were happy to be interviewed as no one usually asked them about these issues. Other participants commented that doing the social atom helped the interviewee stand apart from her problem and look at it, and that doing the “hand” exercise helped people identify their personal resources.

## 7. Conclusion

Work in Vanuatu as part of WHO PIMHnet has shown that equity of access to mental health care is possible when it is integrated into primary care, the workforce is properly trained and mentored, and the Government supports the process. As with many LAMICs, the high need for mental health care in the Western Pacific region was not being met because of lack of workforce, lack of culturally appropriate and practical training, limited financial resources, stigma, and the isolation of many communities. Training the primary health care workforce has begun to address this inequity. With the support of WHO and NZAid, further visits to Vanuatu will support those already trained and begin to target those who can be trainers and mentors themselves. Similar activities are happening in other Pacific Islands. It is the beginning of a new era in mental health care in Pacific Island countries.
